# YEASTRACT+: a portal for the exploitation of global transcription regulation and metabolic model data in yeast biotechnology and pathogenesis

**DOI:** 10.1093/nar/gkac1041

**Published:** 2022-11-09

**Authors:** Miguel Cacho Teixeira, Romeu Viana, Margarida Palma, Jorge Oliveira, Mónica Galocha, Marta Neves Mota, Diogo Couceiro, Maria Galhardas Pereira, Miguel Antunes, Inês V Costa, Pedro Pais, Carolina Parada, Claudine Chaouiya, Isabel Sá-Correia, Pedro Tiago Monteiro

**Affiliations:** Department of Bioengineering, Instituto Superior Técnico, Universidade de Lisboa, Av. Rovisco Pais, 1049-001 Lisbon, Portugal; iBB-Institute for BioEngineering and Biosciences, Biological Sciences Research Group, Av. Rovisco Pais, 1049-001 Lisbon, Portugal; Associate Laboratory i4HB—Institute for Health and Bioeconomy at Instituto Superior Técnico, Universidade de Lisboa, Av. Rovisco Pais, 1049-001 Lisbon, Portugal; Department of Bioengineering, Instituto Superior Técnico, Universidade de Lisboa, Av. Rovisco Pais, 1049-001 Lisbon, Portugal; iBB-Institute for BioEngineering and Biosciences, Biological Sciences Research Group, Av. Rovisco Pais, 1049-001 Lisbon, Portugal; Associate Laboratory i4HB—Institute for Health and Bioeconomy at Instituto Superior Técnico, Universidade de Lisboa, Av. Rovisco Pais, 1049-001 Lisbon, Portugal; Department of Bioengineering, Instituto Superior Técnico, Universidade de Lisboa, Av. Rovisco Pais, 1049-001 Lisbon, Portugal; iBB-Institute for BioEngineering and Biosciences, Biological Sciences Research Group, Av. Rovisco Pais, 1049-001 Lisbon, Portugal; Associate Laboratory i4HB—Institute for Health and Bioeconomy at Instituto Superior Técnico, Universidade de Lisboa, Av. Rovisco Pais, 1049-001 Lisbon, Portugal; INESC-ID, R. Alves Redol, 9, 1000-029 Lisbon, Portugal; Department of Bioengineering, Instituto Superior Técnico, Universidade de Lisboa, Av. Rovisco Pais, 1049-001 Lisbon, Portugal; iBB-Institute for BioEngineering and Biosciences, Biological Sciences Research Group, Av. Rovisco Pais, 1049-001 Lisbon, Portugal; Associate Laboratory i4HB—Institute for Health and Bioeconomy at Instituto Superior Técnico, Universidade de Lisboa, Av. Rovisco Pais, 1049-001 Lisbon, Portugal; Department of Bioengineering, Instituto Superior Técnico, Universidade de Lisboa, Av. Rovisco Pais, 1049-001 Lisbon, Portugal; iBB-Institute for BioEngineering and Biosciences, Biological Sciences Research Group, Av. Rovisco Pais, 1049-001 Lisbon, Portugal; Associate Laboratory i4HB—Institute for Health and Bioeconomy at Instituto Superior Técnico, Universidade de Lisboa, Av. Rovisco Pais, 1049-001 Lisbon, Portugal; Department of Bioengineering, Instituto Superior Técnico, Universidade de Lisboa, Av. Rovisco Pais, 1049-001 Lisbon, Portugal; iBB-Institute for BioEngineering and Biosciences, Biological Sciences Research Group, Av. Rovisco Pais, 1049-001 Lisbon, Portugal; Associate Laboratory i4HB—Institute for Health and Bioeconomy at Instituto Superior Técnico, Universidade de Lisboa, Av. Rovisco Pais, 1049-001 Lisbon, Portugal; Department of Bioengineering, Instituto Superior Técnico, Universidade de Lisboa, Av. Rovisco Pais, 1049-001 Lisbon, Portugal; Department of Bioengineering, Instituto Superior Técnico, Universidade de Lisboa, Av. Rovisco Pais, 1049-001 Lisbon, Portugal; iBB-Institute for BioEngineering and Biosciences, Biological Sciences Research Group, Av. Rovisco Pais, 1049-001 Lisbon, Portugal; Associate Laboratory i4HB—Institute for Health and Bioeconomy at Instituto Superior Técnico, Universidade de Lisboa, Av. Rovisco Pais, 1049-001 Lisbon, Portugal; Department of Bioengineering, Instituto Superior Técnico, Universidade de Lisboa, Av. Rovisco Pais, 1049-001 Lisbon, Portugal; iBB-Institute for BioEngineering and Biosciences, Biological Sciences Research Group, Av. Rovisco Pais, 1049-001 Lisbon, Portugal; Associate Laboratory i4HB—Institute for Health and Bioeconomy at Instituto Superior Técnico, Universidade de Lisboa, Av. Rovisco Pais, 1049-001 Lisbon, Portugal; Department of Bioengineering, Instituto Superior Técnico, Universidade de Lisboa, Av. Rovisco Pais, 1049-001 Lisbon, Portugal; iBB-Institute for BioEngineering and Biosciences, Biological Sciences Research Group, Av. Rovisco Pais, 1049-001 Lisbon, Portugal; Associate Laboratory i4HB—Institute for Health and Bioeconomy at Instituto Superior Técnico, Universidade de Lisboa, Av. Rovisco Pais, 1049-001 Lisbon, Portugal; Department of Bioengineering, Instituto Superior Técnico, Universidade de Lisboa, Av. Rovisco Pais, 1049-001 Lisbon, Portugal; Aix Marseille Univ, CNRS, I2M, Marseille, France; Department of Bioengineering, Instituto Superior Técnico, Universidade de Lisboa, Av. Rovisco Pais, 1049-001 Lisbon, Portugal; iBB-Institute for BioEngineering and Biosciences, Biological Sciences Research Group, Av. Rovisco Pais, 1049-001 Lisbon, Portugal; Associate Laboratory i4HB—Institute for Health and Bioeconomy at Instituto Superior Técnico, Universidade de Lisboa, Av. Rovisco Pais, 1049-001 Lisbon, Portugal; Department of Computer Science and Engineering, Instituto Superior Técnico (IST), Universidade de Lisboa, Av. Rovisco Pais, 1049-001 Lisbon, Portugal; INESC-ID, R. Alves Redol, 9, 1000-029 Lisbon, Portugal

## Abstract

YEASTRACT+ (http://yeastract-plus.org/) is a tool for the analysis, prediction and modelling of transcription regulatory data at the gene and genomic levels in yeasts. It incorporates three integrated databases: YEASTRACT (http://yeastract-plus.org/yeastract/), PathoYeastract (http://yeastract-plus.org/pathoyeastract/) and NCYeastract (http://yeastract-plus.org/ncyeastract/), focused on *Saccharomyces cerevisiae*, pathogenic yeasts of the *Candida* genus, and non-conventional yeasts of biotechnological relevance. In this release, YEASTRACT+ offers upgraded information on transcription regulation for the ten previously incorporated yeast species, while extending the database to another pathogenic yeast, *Candida auris*. Since the last release of YEASTRACT+ (January 2020), a fourth database has been integrated. CommunityYeastract (http://yeastract-plus.org/community/) offers a platform for the creation, use, and future update of YEASTRACT-like databases for any yeast of the users’ choice. CommunityYeastract currently provides information for two *Saccharomyces boulardii* strains, *Rhodotorula toruloides* NP11 oleaginous yeast, and *Schizosaccharomyces pombe* 972h-. In addition, YEASTRACT+ portal currently gathers 304 547 documented regulatory associations between transcription factors (TF) and target genes and 480 DNA binding sites, considering 2771 TFs from 11 yeast species. A new set of tools, currently implemented for *S. cerevisiae* and *C. albicans*, is further offered, combining regulatory information with genome-scale metabolic models to provide predictions on the most promising transcription factors to be exploited in cell factory optimisation or to be used as novel drug targets. The expansion of these new tools to the remaining YEASTRACT+ species is ongoing.

## INTRODUCTION

Yeasts are a diverse group of unicellular fungal species with a strong impact on human life. The most well-known yeast is by far *Saccharomyces cerevisiae*, long used unknowingly for its alcoholic fermentation ability in the brewer and wine industries, but also in the production of bread and other dough-based products. Given its early biotechnological success, its genetic amenability and its genome fully sequenced since 1996 (1), *S. cerevisiae* has been exploited as a cell factory for the industrial production of many added-value compounds (2). Recent years have seen a tremendous increase in the number and variety of yeast species displaying a biotechnological potential thanks to their natural properties. Among them are the methylotrophic yeast *Komagataella phaffii* (formerly *Pichia pastoris*), a favourite host for recombinant protein production (3); the weak acid-resistant food spoilage yeast *Zygosaccharomyces baillii* (4); *Kluyveromyces lactis*, widely used in cheese production (5); the thermotolerant yeast *Kluyveromyces marxianus* (6) and the oleaginous yeast *Yarrowia lipolytica* (7).

On the other end of the spectrum lay pathogenic yeasts of the *Candida* genus, major causative agents of human systemic fungemia, and responsible for more than 400,000 in life-threatening infections worldwide every year (8). *Candida albicans*, *Candida glabrata*, *Candida parapsilosis* and *Candida tropicalis* are the most prevalent among candidiasis patients, accounting for >90% of all *Candida* infections (9). More recently, *Candida auris* arose as a pathogen of concern, being associated with the first cases of candidiasis outbreaks in hospital environments, and displaying unusual resistance to the currently available antifungal armamentarium (10).

A complete understanding of the molecular and regulatory mechanisms that control the productivity in biotechnologically-relevant yeasts is key to guiding the design of more effective cell factories. Simultaneously, understanding the molecular mechanisms that control phenotypes related to pathogenesis in human pathogens is essential to guide the design of more effective therapeutic options. One of the most promising Systems Biology based methodologies to address both issues is the use of Genome-Scale Metabolic Models (GSMMs), which provide a simplified, yet comprehensive, view of the full metabolism of an organism, and enable the simulation of the system’s behaviour. Indeed, metabolic engineering based on GSMMs has been successful in optimising the production of added-value compounds in yeasts (11). In parallel, GSMMs have also been exploited in the search for promising new drug targets, by facilitating the prediction of gene essentiality in pathogenic organisms (12). However, the lack of integration of regulatory information in the currently available GSMMs hinders their predictive ability, preventing the ability to identify transcription factors as promising targets for metabolic engineering or for the design of new antifungal drugs.

In this release, the most recent YEASTRACT+ upgrade is presented, including up-to-date curated information on all published regulatory associations between transcription factors (TFs) and target genes or TFs and their DNA binding sites. Besides the previously integrated YEASTRACT (13–18), PathoYeastract (19) and NCYeastract (20) databases, it also presents upgrades in three dimensions: (i) the introduction of a fourth database, CommunityYeastract; (ii) the integration of *C. auris*, another *Candida* species in the PathoYeastract database and (iii) a set of new computational tools, that combine regulatory data with genome-scale metabolic models, aiming the prediction of the most promising TFs to be exploited in cell factory or to be used as novel drug targets.

## DATA UPDATE AND UPGRADE

In this paper, the upgrade of the YEASTRACT+ portal is presented, including updates on the YEASTRACT, PathoYeastract and NCYeastract databases, as detailed in Table 1.

**Table 1.** Number of transcription factors (TFs) with regulatory associations, number of regulatory associations between TFs and target genes (TGs), as well as the number of TF binding sites (TFBSs) for the yeast species of the YEASTRACT+ databases

Yeast	# TFs	# TF-TG associations	# TFBSs
YEASTRACT			
*Saccharomyces cerevisiae*	226	215 398	2 264
NCYeastract			
*Komagataella phaffii*	14	6 434	1
*Zygosaccharomyces baillii*	1	47	2
*Kluyveromyces lactis*	17	313	2
*Kluyveromyces marxianus*	2	1 148	0
*Yarrowia lipolytica*	7	9 874	2
PathoYeastract			
*Candida albicans*	129	51 224	93
*Candida glabrata*	50	10 101	42
*Candida parapsilosis*	12	7 120	6
*Candida tropicalis*	18	2 881	1
*Candida auris*	4	7	0

YEASTRACT, focused on *S. cerevisiae*, currently includes 215 398 regulatory associations between TFs and target genes, as well as 310 associations between TFs and TF binding sites, which corresponds to a 5% increase in the amount of available data since its latest release. Data on transcriptional regulatory associations in NCYeastract was also updated. Specifically, 1%, 0%, 4.3%, 0.9% and 0.5% increases in the number of regulatory associations between TFs and target genes, experimentally determined in *Komagataella phaffii*, *Zygosaccharomyces baillii*, *Kluyveromyces lactis*, *Kluyveromyces marxianus* and *Yarrowia lipolytica*, respectively, were registered in the last 2 years. In the case of PathoYeastract, the number of regulatory associations between TFs and target genes deposited in the database increased 2%, 114%, 0% and 0.4% for *C. albicans*, *C. glabrata*, *C. parapsilosis* and *C. tropicalis*, respectively. Additionally, a fifth species of pathogenic yeast was included in the database, *C. auris*. Despite the fact that relatively little is yet known about this emergent species, its predicted impact on the clinical development of recalcitrant candidiasis, associated with hospital outbreaks of the disease, led us to provide the community with this resource, which currently includes only seven experimentally characterised associations between TFs and target genes.

All TF–target gene and TF-TF binding site associations deposited in YEASTRACT+ are provided with specific information on the underlying publication, the experimental setup used to identify each regulatory association, including classification of the used approach as either based on DNA binding (e.g. Chromatin ImmunoPrecipitation (ChIP), ChIP-on-chip, ChIP-seq and Electrophoretic Mobility Shift Assay (EMSA)) or Expression (e.g. RT-PCR, microarray hybridisation, RNA sequencing or expression proteomics) data, as well as information on the environmental conditions in which each association was found to take place.

Altogether, YEASTRACT+ gathers a total of 304 547 documented regulatory associations between transcription factors (TFs) and target genes and 2,771 DNA binding sites, considering 480 TFs in the 11 yeast species. Also, 276 389 Gene Ontology (GO) terms (21), associated with the compiled yeast genes, are currently gathered in the database, from the gene association data provided by SGD (http://sgd-archive.yeastgenome.org/curation/literature/) (22), CGD (http://www.candidagenome.org/download/go/) (23) and PomBase (https://www.pombase.org/downloads/go-annotations) (24).

The increasing exploitation of a variety of yeast species of biotechnology or medical interest constitutes a challenge, as many of them are poorly characterised, particularly in terms of their transcriptional networks. The lack of data in these organisms, especially when compared with the model yeast *S. cerevisiae*, can, at least partially, be compensated by the use of comparative genomics approaches. These permit the exploitation of the knowledge of well-known organisms to predict the function and regulation of orthologous proteins in poorly characterised or uncharacterised systems. Naturally, given that the conservation of gene and TF function, TF binding sites and regulatory associations among different species is not complete, results obtained through this comparative genomics approach should be regarded as merely indicative, requiring experimental validation. Still, with this in mind, the possibility of expanding YEASTRACT+ to an unlimited number of yeast species, for which no specific regulatory data is gathered, but whose genomic sequence can be used to predict gene and genome-wide regulatory pathways, led to the development of CommunityYeastract.

CommunityYeastract (Community Yeast Search for Transcriptional Regulators And Consensus Tracking) is a repository of automatically generated YEASTRACT-like databases, for yeast species or strains, according to the request of community members (20). No data on transcription associations documented for the specific organism is included. However, all YEASTRACT+ queries may be run on genes or datasets of the specific organism, considering regulatory information of homologous genes in related yeast species fully described in YEASTRACT, PathoYeastract and NCYeastract.

CommunityYeastract currently provides information for two probiotic *Saccharomyces boulardii* strains, Biocodex and Unique 28 (25), the oleaginous yeast *Rhodotorula toruloides* NP11 (26), and the model fission yeast *Schizosaccharomyces pombe* 972h-. Tools to automatically generate YEASTRACT-like databases, based on genome sequences, were provided elsewhere (26). However, the YEASTRACT team welcomes requests from its users or potential users to add additional yeast species to CommunityYeastract.

## INTEGRATION OF GENOME-SCALE METABOLIC MODELS WITH REGULATORY INFORMATION: NEW TOOLS FOR STRAIN OPTIMISATION AND DRUG TARGET IDENTIFICATION

Genome-Scale Metabolic Models (GSMMs) aim to provide a reconstruction of the whole metabolism of an organism, through its description as a mathematical model. The first GSMM was built for *Haemophilus influenzae*, in 1999 (27), followed by *Escherichia coli*, in 2000 (28), and by *S. cerevisiae*, in 2003 (29). Throughout the last two decades, numerous GSMMs have been constructed, including some dedicated to multicellular organisms, including humans (30). GSMMs contain three main levels of information: metabolites, reactions and metabolic genes. The relationships between metabolites and reactions can be described by a stoichiometric matrix and the ones between reactions and genes by a binary matrix. A well-constructed model enables the simulation of an organism’s behaviour - i.e. how much of each metabolite is produced or consumed—in a given medium/environmental condition, all done *in silico*, with constraint-based modelling (30). Despite many efforts for integrating omics data, including transcriptomics, proteomics, metabolomics and fluxomics data, into available metabolic models, it is still not possible to integrate full regulatory data in any of the currently available metabolic models.

In this YEASTRACT+ release, automated tools to exploit current yeast GSMMs are provided for *S. cerevisiae* and *C. albicans*, relying on COBRApy (31). The expansion of their use to all other yeasts in the database is envisaged. Two main goals can now be achieved using the proposed new queries: (i) the prediction of the genes whose expression manipulation may lead to increased production of a chosen metabolite, in a metabolic engineering perspective and (ii) the prediction of the genes that may be used as drug targets, based on their essentiality in chosen conditions. Thanks to the integration of regulatory information, it is also possible to predict the TFs whose expression is worth manipulating to optimise metabolite production or the TFs that may be considered promising drug targets. Details on how these new tools can be used in these contexts, follow.

### Prediction of metabolic and TF encoding genes envisaging cell factory optimisation

Using the new YEASTRACT query ‘Predict [metabolite] optimisation by manipulating gene expression’, it is possible to search for the genes whose deletion, down-regulation or up-regulation is predicted to improve the production of a metabolite of interest. The *S. cerevisiae* GSMM model currently used in YEASTRACT+ is Yeast8 (32). The query includes the selection of a specific growth medium. Currently, two growth media are available - Synthetic Minimal medium and Glucose-rich Synthetic Complete medium - whose compositions are shown by clicking the link ‘model/medium’. Aiming the optimisation of the production of a chosen compound, genes or TFs predicted to be of interest can be ranked according to one of the three criteria: ‘Reaction flux’, ‘Biomass-Product Coupled Yield (BPCY)’ or ‘Product Yield with Minimum Biomass (PYMB)’.

**Figure 1.**
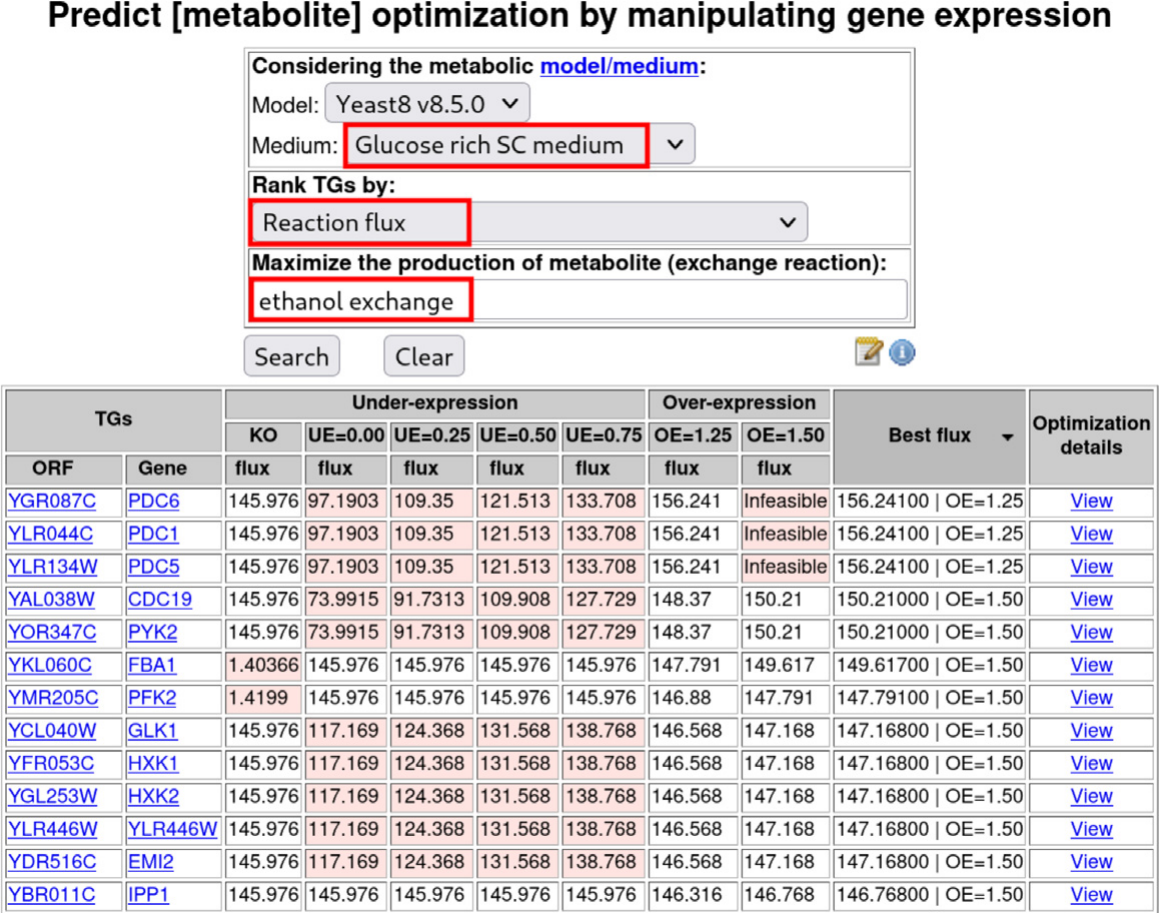
Depiction of the query ‘Predict [metabolite] optimisation by manipulating gene expression’. Top: set of options with the selected medium, rank criterion and metabolite highlighted in red. Bottom: table listing the genes whose expression manipulation is predicted to optimise the production of the selected metabolite, obtained by simulating the selected GSMM and the selected medium. Here, the predicted production is given in terms of the exchange reaction flux. The impact on the metabolite production of various gene expression manipulations is displayed, from full gene Knock Out (KO) or decreased expression (UE, Under-Expression, from 0 to 0.75-fold the wild-type levels), to increased expression (OE, Over-Expression, from 1.25- to 1.5-fold the wild-type levels). Cells shaded in salmon contain values lower than the WT exchange reaction flux or infeasible cases. The last two columns highlight the highest metabolite production and associated manipulation of gene expression, followed by the ‘View’ link for details on the changes imposed on reaction fluxes by said manipulation.

For example, to identify genes whose expression manipulation may increase ethanol production in *S. cerevisiae*, ‘Glucose-rich SC medium’ is selected, as it mimics a situation of high glucose availability and low oxygen availability, which is typical of industrial alcoholic fermentation (Figure 1). The metabolite of interest is defined in the appropriate box ‘ethanol exchange’. Upon clicking the ‘Search’ button, the results are displayed in a table format, listing the genes whose expression manipulation is predicted to optimise ethanol production, in the pre-selected conditions (Figure 1). Predicted metabolite production is given in terms of metabolite exchange flux. The impact on metabolite production of different changes in gene expression is displayed in the table, from full gene Knock Out (KO) or decreased gene expression (UE, Under-Expression, from 0 to 0.75-fold the wild-type levels), to increased gene expression (OE, Over-Expression, from 1.25- to 1.5-fold the wild-type levels) (33). The final columns highlight the highest level of metabolite production with the manipulation leading to that level, followed by the ‘View’ link, which allows obtaining details on the changes imposed on reaction fluxes by said manipulation. In this case, the over-expression of 67 genes or the deletion/down-regulation of 106 genes is expected to result in a moderate increase in ethanol production. For example, increasing the expression of *PDC1*, *PDC5* or *PDC6*, encoding three pyruvate decarboxylases, is predicted to increase ethanol production, possibly by increasing the production of acetaldehyde, which may then be converted into ethanol by alcohol dehydrogenases. Another suggested route for increased ethanol production is the deletion of any one of the 18 ATP genes, encoding subunits of the F1F0-ATP synthase that catalyse the last step of oxidative phosphorylation, which requires the consumption of ethanol or ethanol precursors, through respiration.

**Figure 2.**
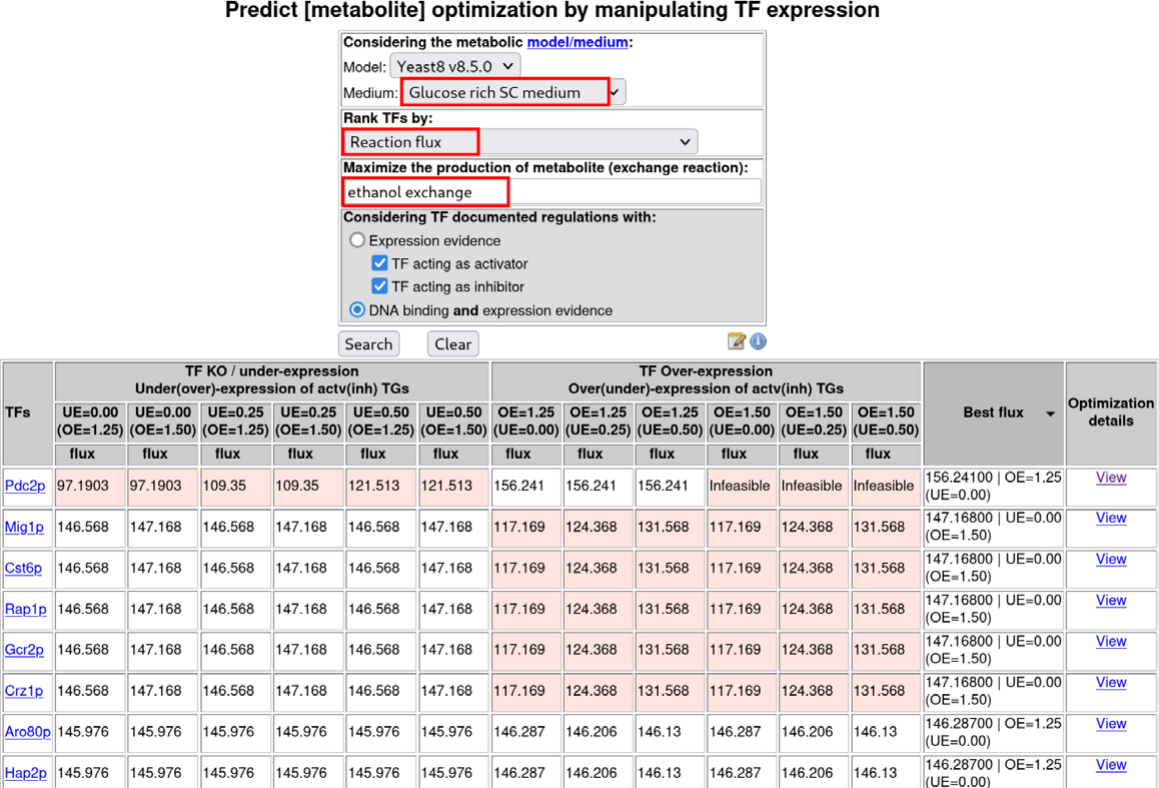
Depiction of the query ‘Predict [metabolite] optimisation by manipulating TF expression’. Top: set of options with the selected medium, rank criterion and metabolite exchange reaction highlighted in red. Bottom: corresponding table of results listing the TFs whose expression manipulation is predicted to optimise the production of the selected metabolite, obtained by simulating the selected GSMM and the selected medium. Here, the predicted production is given in terms of the exchange reaction flux. The impact on the metabolite production of TF Knock Out (KO) or Over-Expression (OE) is displayed, for different effects of the TF expression manipulation on its target genes (TGs). A TF Knock Out (KO) effect ranges from Under-Expression (UE) of its activated TGs (from 0 to 0.5-fold the wild-type levels) to OE of its repressed TGs (from 1.25- to 1.5-fold the wild-type levels). A TF OE effect ranges from OE of its activated TGs (from 0 to 0.5-fold the wild-type levels) to UE of its repressed TGs (from 1.25- to 1.5-fold the wild-type levels). The last two columns highlight the highest level of the metabolite production and associated manipulations of TGs expression, followed by the ‘View’ link for details on the changes imposed on reaction fluxes by said manipulations.

The most novel outcome of this new set of tools is obtained with the query ‘Predict [metabolite] optimisation by manipulating Transcription Factor (TF) expression’ (Figure 2). This tool enables the identification of the TFs whose deletion, down-regulation or up-regulation is predicted to improve the production of a metabolite of interest. Here again, the user may choose ‘Glucose-rich SC medium’, and ‘ethanol exchange’ as the reaction to be optimised. It is possible to filter the regulations to be considered, selecting documented regulations with expression evidence, positive and/or negative, or additionally requiring DNA binding evidence. Once the ‘Search’ button is clicked, the results are displayed in a table listing the TFs whose expression manipulation is predicted to enable the optimisation of ethanol production, in the pre-selected conditions (Figure 2). Predicted metabolite production is given in terms of metabolite exchange flux. The impact on metabolite production of TF KO or OE is predicted, considering a wide range of possible effects of the TF on the expression of its activated and repressed target genes (UE, Under-Expression, of TF activated target genes from 0 to 0.5-fold the wild-type levels; OE, Over-Expression, of TF repressed target genes from 1.25 to 1.5-fold the wild-type levels). The final columns highlight the highest level of the metabolite production obtained by the expression manipulation of each TF, followed by the possibility to ‘View’ details on the changes imposed on reaction fluxes by said manipulations. In this case, the over-expression of 18 TFs or the deletion of 23 TFs is expected to result in a moderate increase in ethanol production. For example, increasing the expression of *PDC2* TF encoding gene is predicted to increase ethanol production. Interestingly, Pdc2 controls the expression of *PDC1* and *PDC5*, whose own over-expression is predicted to increase ethanol production, as discussed above. On the other hand, the KO of *MIG1*, *GCR2* or *HAP2*, encoding TFs involved in the control of glucose repression, glycolysis and respiration, respectively, are predicted to lead to increased ethanol production, likely through their effect on the expression of a combination of central carbon metabolism genes. As far as our knowledge goes, the impact of the expression level of these TFs on ethanol production has never been evaluated.

If the user wishes to use a growth medium or a yeast model that is not currently available at YEASTRACT, (s)he is invited to contact our support team to evaluate its importance and to make it available to the wider community.

### Prediction of metabolic and TF encoding genes as promising drug targets

The new ‘Essentiality’ prediction query is offered to YEASTRACT+ users, particularly with the aim of identifying new drug targets. The use of this new tool can be exemplified in the case of the human pathogen *C. albicans*. The *C. albicans* GSMM model currently used by YEASTRACT is iRV781 (34). The query includes the selection of a specific growth medium. Currently, two growth media are available—Synthetic Minimal Medium and RPMI 1640 medium—whose compositions are shown by clicking the link ‘model/medium’. The essentiality search can be performed by looking for essential genes, essential reactions (which may be coupled to several metabolic genes) or essential TFs.

**Figure 3.**
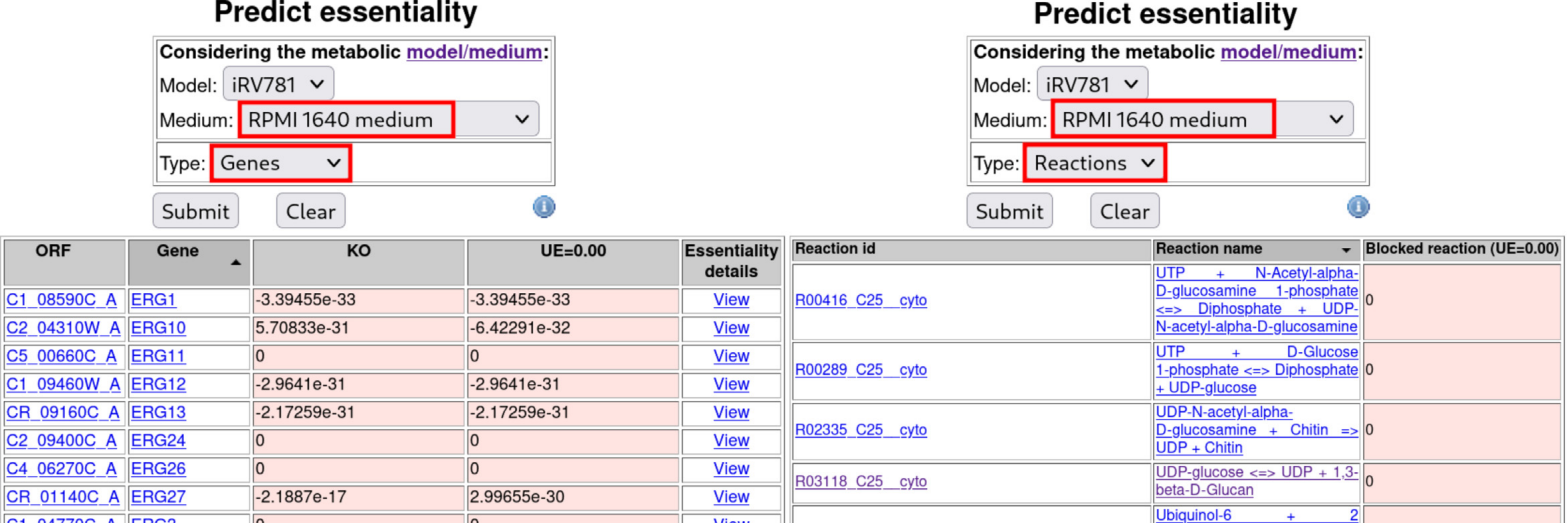
Depiction of the ‘Essentiality’ prediction query for *Candida albicans*. Top: set of options with the selected medium, and whether essentiality is evaluated for genes, reactions or TFs, as highlighted in red rectangles. Bottom left: table of results of essential genes for RPMI medium. Predicted essential genes, as defined by COBRApy, are those whose single Knock Out (KO) is predicted to lead to biomass production flux below 1% of that of the wild-type strain. The biomass production flux predicted upon *in silico* deletion of the indicated gene/ORF is displayed. Although KO and UE=0.0 (Under-Expression) appear to be the same, the simulation tools handle them differently for reactions having multiple enzymes with the same function. In such a case, the gene ‘KO’ is simulated as having no impact on reaction flux (the other isoenzymes are supposed to fully replace the deleted one), while ‘UE=0.0’ is simulated by a decrease of the reaction flux, inversely proportional to the number of isoenzymes (e.g. if 3 isoenzymes catalyse a reaction, the deletion of one of the coding genes will lead to a 33% reduction of the reaction flux). Bottom right: predicted essential reactions for RPMI medium, as defined by COBRApy, that is those whose blockage (reaction flow = 0) leads to biomass production flux below 1% of that of the wild-type strain. The predicted biomass production flux is displayed together with the reaction ID/and name.

For example, if the user wishes to identify *C. albicans* metabolic genes, which are essential under conditions found in the human host environment, ‘RPMI 1640 medium’ may be selected as it mimics human serum (Figure 3). Upon selecting ‘Genes’ and once the ‘Search’ button is clicked, the results are displayed in a table format, listing the genes whose deletion leads to biomass production flux below 1% of that of the wild-type strain, in the selected growth medium (Figure 3). Consistent with the proposed applicability of this approach, among the list of identified essential genes are ergosterol biosynthesis genes, including *ERG11*, which encodes the target of the currently used family of azole antifungal drugs, as reviewed in (35). Remarkably, the *GSC1* gene, encoding the target of echinocandin antifungal drugs, is not identified as an essential gene, in this query. The reason for this is that in *C. albicans* there are two paralogs of *GSC1*, *GSL1* and *GSL2*, which are predicted to maintain cell viability when *GSC1* is absent. For such cases, searching for essential reactions, instead of essential genes, is more promising. When using the ‘Essentiality’ prediction query, selecting ‘Reactions’, the results displayed in a table format, provide the list of reactions whose blockage (reaction flow = 0) is predicted to lead to biomass production flux <1% of those of the wild-type strain, in the selected growth medium. In this list of essential reactions it is possible to detect the reaction ‘UDP-glucose <= > UDP + 1,3-beta-d-Glucan’. If the user follows the link associated with the reaction name, the underlying genes are indicated, which, in this case, include precisely the echinocandin encoding targets *GSC1*, *GSL1* and *GSL2*.

**Figure 4.**
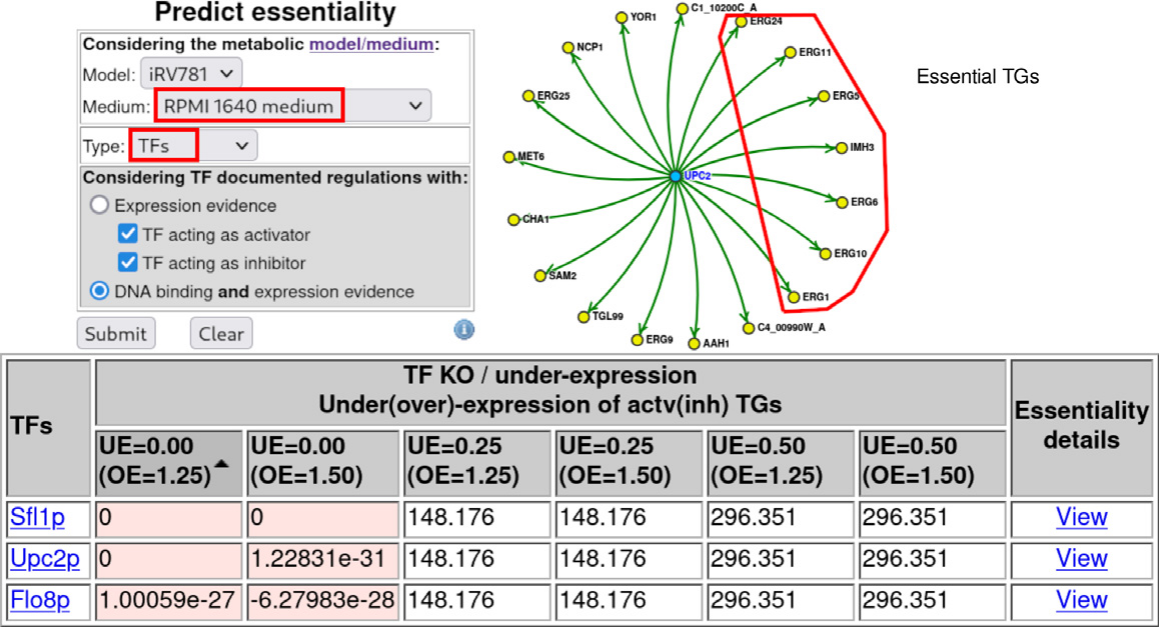
Depiction of the ‘Essentiality’ prediction query when looking for essential TFs in *Candida albicans*. Top left: set of options with the selected medium, and whether essentiality is evaluated for genes, reactions or TFs, as highlighted in red rectangles. Bottom: table of essential TFs for the RPMI medium. Predicted essential TFs, as defined by COBRApy, are those whose single Knock Out leads to biomass production flux below 1% of that of the wild-type strain. The predicted biomass production flux upon *in silico* Knocking Out (KO) or Under-Expression (UE) of each TF is displayed, considering impacts on the expression of its target genes (TGs) ranging from UE activated TGs (from 0 to 0.5-fold the wild-type levels) to Over-Expression (OE) of repressed TGs (from 1.25- to 1.5-fold the wild-type levels). Top right: regulatory network of one of the identified essential TFs, Upc2, and the genes whose expression is controlled by that same TF. This visualisation was obtained by following the corresponding ‘View’ link, in the results table. Highlighted in the red circle are the seven Upc2 TGs predicted to be essential in the same environmental conditions.

Again, the most novel outcome of this new set of tools is obtained with the ‘Essentiality’ prediction query, option ‘TFs’, as it enables the identification of the TFs whose deletion is predicted to lead to biomass production flux below 1% of that of the wild-type strain, in the selected growth medium. Again, the user may choose ‘RPMI 1640 medium’ as the condition of choice. Once the ‘Search’ button is clicked, the table of results lists the TFs predicted to be essential in the pre-selected conditions (Figure 4). Three TFs are predicted to be essential in ‘RPMI 1640 medium’. Although none of them is encoded by a truly essential gene (whose deletion generates an unviable cell), the YEASTRACT+ modelling tools predict that in this medium, mimicking human serum, they are crucial for biomass production. Although the exact effect of TF deletion in the metabolic reaction fluxes is difficult to predict, it is interesting to observe, in Figure 4, that, for example, the Upc2 TF does indeed control the expression of 18 metabolic genes, seven of them being involved in ergosterol biosynthesis and predicted to be essential in the same environmental conditions. This result is consistent with *UPC2* essentiality in these conditions.

## FUTURE DIRECTIONS

The YEASTRACT+ team is committed to continuous update, and offer reliable and complete information on yeast transcription regulation to the international research community. As the scope of the database is expanded to cover a wider range of yeast species of biotechnological or medical interest, made easier with the creation of CommunityYeastract, it is expected that the ability to serve better our users increases. The expansion of the new network modelling tools to all yeast species for which a GSMM is available will be pursued, as well as the increase in the number of options offered in this context, particularly the possibility to predict synthetic lethality as a means to identify possible targets for combination therapy.

## DATA AVAILABILITY

All data underlying this article are available through the YEASTRACT+ portal without restrictions (http://yeastract-plus.org/). Flat files for computational analyses are shared on request.

## References

[1] Goffeau A. , Barrell B.G., Bussey H., Davis R.W., Dujon B., Feldmann H., Galibert F., Hoheisel J.D., Jacq C., Johnston M. et al. Life with 6000 Genes. Science. 1996; 274:546–567. 8849441 10.1126/science.274.5287.546

[2] Li M. , Borodina I. Application of synthetic biology for production of chemicals in yeast *Saccharomyces cerevisiae*. FEMS Yeast Res. 2014; 15:1–12. 10.1111/1567-1364.12213 25238571

[3] Gasser B. , Mattanovich D. A yeast for all seasons – is *Pichia pastoris* a suitable chassis organism for future bioproduction. FEMS Microbiol. Lett. 2018; 365:10.1093/femsle/fny181. 30052876

[4] Palma M. , Guerreiro J.F., Sá-Correia I. Adaptive response and tolerance to acetic acid in *Saccharomyces cerevisiae* and *Zygosaccharomyces bailii*: a physiological genomics perspective. Front. Microbiol. 2018; 9:274. 29515554 10.3389/fmicb.2018.00274 PMC5826360

[5] Spohner S.C. , Schaum V., Quitmann H., Czermak P. Kluyveromyces lactis: an emerging tool in biotechnology. J. Biotech. 2016; 222:104–116. 10.1016/j.jbiotec.2016.02.023 26912289

[6] Cernak P. , Estrela R., Poddar S., Skerker J.M., Cheng Y.-F., Carlson A.K., Chen B., Glynn V.M., Furlan M., Ryan O.W. et al. Engineering *Kluyveromyces marxianus* as a robust synthetic biology platform host. mBio. 2018; 9:e01410-18. 30254120 10.1128/mBio.01410-18 PMC6156195

[7] Mota M. , Múgica P., Sá-Correia I. Exploring yeast diversity to produce lipid-based biofuels from agro-forestry and industrial organic residues. J. Fungi. 2022; 8:687. 10.3390/jof8070687 PMC9315891 35887443

[8] Wisplinghoff H. , Bischoff T., Tallent S.M., Seifert H., Wenzel R.P., Edmond M.B. Nosocomial bloodstream infections in US hospitals: analysis of 24, 179 cases from a prospective nationwide surveillance study. Clin. Infect. Dis. 2004; 39:309–317. 15306996 10.1086/421946

[9] Guinea J. Global trends in the distribution of *Candida* species causing candidemia. Clin. Microbiol. Infect. 2014; 20:5–10. 10.1111/1469-0691.12539 24506442

[10] Worku M. , Girma F. *Candida auris*: from multidrug resistance to Pan-resistant strains. Infect. Drug Resist. 2020; 13:1287–1294. 32440165 10.2147/IDR.S249864 PMC7211321

[11] Gu C. , Kim G.B., Kim W.J., Kim H.U., Lee S.Y. Current status and applications of genome-scale metabolic models. Genome Biol. 2019; 20:121. 31196170 10.1186/s13059-019-1730-3 PMC6567666

[12] Raškevičius V. , Mikalayeva V., Antanavičiūtė I., Ceslevičienė I., Skeberdis V.A., Kairys V., Bordel S. Genome scale metabolic models as tools for drug design and personalized medicine. PLOS One. 2018; 13:e0190636. 29304175 10.1371/journal.pone.0190636 PMC5755790

[13] Teixeira M.C. The YEASTRACT database: a tool for the analysis of transcription regulatory associations in *Saccharomyces cerevisiae*. Nucleic Acids Res. 2006; 34:D446–D451. 16381908 10.1093/nar/gkj013 PMC1347376

[14] Monteiro P.T. , Mendes N.D., Teixeira M.C., d’Orey S., Tenreiro S., Mira N.P., Pais H., Francisco A.P., Carvalho A.M., Lourenco A.B. et al. YEASTRACT-DISCOVERER: New tools to improve the analysis of transcriptional regulatory associations in *Saccharomyces cerevisiae*. Nucleic Acids Res. 2007; 36:D132–D136. 18032429 10.1093/nar/gkm976 PMC2238916

[15] Abdulrehman D. , Monteiro P.T., Teixeira M.C., Mira N.P., Lourenco A.B., dos Santos S.C., Cabrito T.R., Francisco A.P., Madeira S.C., Aires R.S. et al. YEASTRACT: providing a programmatic access to curated transcriptional regulatory associations in *Saccharomyces cerevisiae* through a web services interface. Nucleic Acids Res. 2010; 39:D136–D140. 20972212 10.1093/nar/gkq964 PMC3013800

[16] Teixeira M.C. , Monteiro P.T., Guerreiro J.F., Gonçalves J.P., Mira N.P., dos Santos S.C., Cabrito T.R., Palma M., Costa C., Francisco A.P. et al. The YEASTRACT database: an upgraded information system for the analysis of gene and genomic transcription regulation in *Saccharomyces cerevisiae*. Nucleic Acids Res. 2013; 42:D161–D166. 24170807 10.1093/nar/gkt1015 PMC3965121

[17] Teixeira M.C. , Monteiro P.T., Palma M., Costa C., Godinho C.P., Pais P., Cavalheiro M., Antunes M., Lemos A., Pedreira T. et al. YEASTRACT: An upgraded database for the analysis of transcription regulatory networks in *Saccharomyces cerevisiae*. Nucleic Acids Res. 2017; 46:D348–D353. 10.1093/nar/gkx842 PMC5753369 29036684

[18] Monteiro P.T. , Oliveira J., Pais P., Antunes M., Palma M., Cavalheiro M., Galocha M., Godinho C.P., Martins L.C., Bourbon N. et al. YEASTRACT+: a portal for cross-species comparative genomics of transcription regulation in yeasts. Nucleic Acids Res. 2019; 48:D642–D649. 10.1093/nar/gkz859 PMC6943032 31586406

[19] Monteiro P.T. , Pais P., Costa C., Manna S., Sá-Correia I., Teixeira M.C. The PathoYeastract database: an information system for the analysis of gene and genomic transcription regulation in pathogenic yeasts. Nucleic Acids Res. 2016; 45:D597–D603. 27625390 10.1093/nar/gkw817 PMC5210609

[20] Godinho C.P. , Palma M., Oliveira J., Mota M.N., Antunes M., Teixeira M.C., Monteiro P.T., Sá-Correia I. The N.C.Yeastract and CommunityYeastract databases to study gene and genomic transcription regulation in non-conventional yeasts. FEMS Yeast Res. 2021; 21:foab045. 34427650 10.1093/femsyr/foab045

[21] Carbon S. , Ireland A., Mungall C.J., Shu S., Marshall B., Lewis S. AmiGO: online access to ontology and annotation data. Bioinformatics. 2009; 25:288–289. 19033274 10.1093/bioinformatics/btn615 PMC2639003

[22] Engel S.R. , Dietrich F.S., Fisk D.G., Binkley G., Balakrishnan R., Costanzo M.C., Dwight S.S., Hitz B.C., Karra K., Nash R.S. et al. The Reference Genome Sequence of Saccharomyces cerevisiae: Then and Now. G3: Genes Genomes Genetics. 2014; 4:389–398. 24374639 10.1534/g3.113.008995 PMC3962479

[23] Skrzypek M.S. , Binkley J., Binkley G., Miyasato S.R., Simison M., Sherlock G. The Candida Genome Database (CGD): incorporation of Assembly 22, systematic identifiers and visualization of high throughput sequencing data. Nucleic Acids Res. 2017; 45:D592–D596. 27738138 10.1093/nar/gkw924 PMC5210628

[24] Harris M.A. , Rutherford K.M., Hayles J., Lock A., Bähler J., Oliver S.G., Mata J., Wood V. Fission stories: using PomBase to understand Schizosaccharomyces pombe biology. Genetics. 2021; 220:iyab222. 10.1093/genetics/iyab222 PMC9209812 35100366

[25] Pais P. , Oliveira J., Almeida V., Yilmaz M., Monteiro P.T., Teixeira M.C. Transcriptome-wide differences between Saccharomyces cerevisiae and Saccharomyces cerevisiae var. boulardii: Clues on host survival and probiotic activity based on promoter sequence variability. Genomics. 2021; 113:530–539. 33482324 10.1016/j.ygeno.2020.11.034

[26] Oliveira J. , Antunes M., Godinho C.P., Teixeira M.C., Sá-Correia I., Monteiro P.T. From a genome assembly to full regulatory network prediction: the case study of Rhodotorula toruloides putative Haa1-regulon. BMC Bioinformatics. 2021; 22:399. 34376148 10.1186/s12859-021-04312-3 PMC8353774

[27] Edwards J.S. , Palsson B.O. Systems properties of the *Haemophilus influenzae*Rd metabolic genotype. J. Biol. Chem. 1999; 274:17410–17416. 10364169 10.1074/jbc.274.25.17410

[28] Edwards J.S. , Palsson B.O. The *Escherichia coli* MG1655 in silico metabolic genotype: its definition, characteristics, and capabilities. Proc. Natl. Acad. Sci. U.S.A. 2000; 97:5528–5533. 10805808 10.1073/pnas.97.10.5528 PMC25862

[29] Förster J. , Famili I., Fu P., Palsson B.Ø., Nielsen J. Genome-scale reconstruction of the *Saccharomyces cerevisiae* metabolic network. Genome Res. 2003; 13:244–253. 12566402 10.1101/gr.234503 PMC420374

[30] Zhang C. , Hua Q. Applications of genome-scale metabolic models in biotechnology and systems medicine. Front. Physiol. 2016; 6:413. 26779040 10.3389/fphys.2015.00413 PMC4703781

[31] Ebrahim A. , Lerman J.A., Palsson B.O., Hyduke D.R. COBRApy: COnstraints-Based Reconstruction and Analysis for Python. BMC Syst. Biol. 2013; 7:74. 23927696 10.1186/1752-0509-7-74 PMC3751080

[32] Lu H. , Li F., Sánchez B.J., Zhu Z., Li G., Domenzain I., Marcišauskas S., Anton P.M., Lappa D., Lieven C. et al. A consensus *S. cerevisiae* metabolic model Yeast8 and its ecosystem for comprehensively probing cellular metabolism. Nat. Commun. 2019; 10:3586. 31395883 10.1038/s41467-019-11581-3 PMC6687777

[33] Gonçalves E. , Pereira R., Rocha I., Rocha M. Optimization approaches for the in silico discovery of optimal targets for gene over/underexpression. J. Comput. Biol. 2012; 19:102–114. 22300313 10.1089/cmb.2011.0265

[34] Viana R. , Dias O., Lagoa D., Galocha M., Rocha I., Teixeira M.C. Genome-scale metabolic model of the human pathogen *Candida albicans*: a promising platform for drug target prediction. J. Fungi. 2020; 6:171. 10.3390/jof6030171 PMC7559133 32932905

[35] Pais P. , Galocha M., Teixeira M.C. Genome-wide response to drugs and stress in the pathogenic yeast *Candida glabrata*. Yeasts in Biotechnology and Human Health. 2019; Springer International Publishing 155–193. 10.1007/978-3-030-13035-0_7 30911893

